# Activity in Book-making

**Published:** 1879-04

**Authors:** 


ACTIVITY IN BOOK-MAKING—We have so large a number of recently published books on our table that we have been wholly unable to give them any deserved mention, though we have given our space to that object and made all notices unwarrantably brief. We will review their contents as early as possible, but presume many of our readers will have read and formed their own estimate of their merit before we shall be able to reach them.

